# Advancing amorphous solid dispersions through empirical and hybrid modeling of drug–polymer solubility and miscibility: A case study using Ibuprofen

**DOI:** 10.1016/j.ijpx.2025.100373

**Published:** 2025-08-09

**Authors:** Matheus de Castro, Ana Sara Cordeiro, Mingzhong Li, Christian Lübbert, Catherine McColl, Jatin Khurana, Mark Evans, Walkiria S. Schlindwein

**Affiliations:** a Leicester School of Pharmacy, De Montfort University, Leicester LE1 9BH, United Kingdom; b amofor GmbH, Otto-Hahn-Str. 15, 44227 Dortmund, Germany; c Reckitt Benckiser, Dansom Lane, Hull HU8 7DS, United Kingdom

**Keywords:** Amorphous Solid Dispersion (ASD), Perturbed Chain Statistical Associating Fluid Theory (PC-SAFT), Melting Point Depression (MPD), Phase Diagram, Glass transition

## Abstract

This study investigates the solubility and miscibility of ibuprofen (IBU) with four pharmaceutical polymers, KOLVA64®, KOL17PF®, HPMCAS, and Eudragit® EPO, using a combination of empirical and hybrid modeling approaches, supported by differential scanning calorimetry (DSC) experiments. Traditional group contribution methods based on Hildebrand and Hansen solubility parameters (Fedors, Hoftyzer–van Krevelen, and Just–Breitkreutz) showed variability in solubility predictions but consistently classified all polymer–API blends as miscible (Δδ < 7 MPa^½^). Bagley plots reinforced these findings, although borderline miscibility was indicated for HPMCAS and EPO depending on the method used. A novel attempt to derive the Flory–Huggins (FH) interaction parameter (χ) from solubility parameters at near-melting temperatures showed poor agreement with experimental data, underscoring the limitations of such extrapolations and the semi-empirical nature of the FH model.

Phase diagrams were constructed from DSC-based melting point depression data using three modeling strategies: FH theory, the empirical approach by Kyeremateng (with two fitting methods), and the perturbed-chain statistical associating fluid theory (PC-SAFT) equation of state, both in pure predictions and with fitted binary interaction parameters (k_ij_). The glass transition temperature (T_g_) of the mixtures was modeled using the Gordon–Taylor and Kwei equations. All models provided a consistent polymer ranking based on their solubilizing capacity, with KOL17PF as the most compatible and HPMCAS as the least. Demixing zones (liquid-liquid equilibrium - LLE) predicted by FH and PC-SAFT models suggest that for HPMCAS-based ASDs only very low drug loadings (< 5 % w/w) could potentially be stable at room temperature. In contrast, higher drug loadings (> 10 % w/w) fall under a meta-stable zone with the other polymers, making them better candidates for IBU formulation. HPMCAS also exhibited consistently prediction errors across all T_g_ models, (AARD ∼4.5 %), indicating poorer agreement with experimental data. By integrating empirical and hybrid modeling approaches, this study highlights the strengths and limitations of commonly used solubility prediction methods and advocates for a shift toward a harmonized framework.

## Graphical abstract

**Figure:**
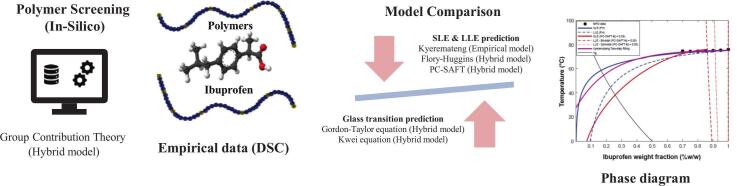

## 1 Introduction

The current pipeline for API development is dominated by poorly soluble compounds, creating a demand for formulation strategies that improve dissolution and bioavailability in oral dosage forms. An effective approach to improve bioavailability is via amorphization, which enhances the active pharmaceutical ingredient's (API) apparent solubility converting it into a higher-energy amorphous state (Li et al., 2019; Mantas et al., 2019). However, despite its advantages in performance, the amorphous form tends to be thermodynamically unstable, naturally reverting to the lower-energy crystalline state (Yu, 2001).

In this context, using polymeric matrices to form amorphous solid dispersions (ASDs) is an effective strategy to enhance both stability and supersaturation behavior during dissolution (Vasconcelos et al., 2016). To ensure long-term stability, it is crucial to understand the miscibility between the active pharmaceutical ingredient (API) and polymer, which is primarily driven by non-covalent interactions, such as van der Waals forces, polar interactions, and hydrogen bonding.

At this point, it is important to differentiate between solubility and miscibility. Solubility refers to the maximum concentration of a solute (e.g., API) that can dissolve in a solvent (typically a polymer in this context) under specific conditions, such as temperature and pressure. In a soluble system, equilibrium is achieved when the solid phase of the API and its dissolved form in the solvent reach a balance (solid-liquid equilibrium, SLE), resulting in a solution where the API is uniformly distributed within the solid polymer matrix.

Miscibility, on the other hand, is the ability of two substances to mix and form a single homogeneous phase, especially in the case of liquids or amorphous materials (liquid-liquid equilibrium, LLE). For an API and a polymer, miscibility refers to the extent to which they can integrate at the molecular level to create a homogeneous amorphous blend. This can be assessed using techniques like differential scanning calorimetry (DSC), where a single glass transition temperature (T_g_) is observed.

Developing ASD-based formulations is a complex process that requires a comprehensive consideration of both formulation and process variables to ensure the production of safe and effective medicines. Quality by Design (QbD), an approach endorsed by the International Council for Harmonization (ICH) and the Food and Drug Administration (FDA), emphasizes a thorough product understanding. This is achieved by defining a Quality Target Product Profile (QTPP) and linking it to Critical Quality Attributes (CQAs), Critical Process Parameters (CPPs), and Critical Material Attributes (CMAs) (ICH Q8(R2), 2009). The Design of Experiments (DoE) methodology is used to conduct optimized experimental studies, allowing for the evaluation of the main effects and interactions among variables, and ultimately building empirical, data-driven models (Butreddy et al., 2021; Triboandas et al., 2024; Bezerra et al., 2025). Despite the explicit benefits, product development within the QbD framework is experimentally driven, thus multiple empirical data must be generated a priori to model building, often constrained by resources (e.g. expensive materials and time) in the industrial setting.

Recent advancements in digitalization, real-time data extraction in pharmaceutical manufacturing, and the transition from batch to continuous processes (Rehrl et al., 2018) have paved the way for Quality by Digital Design (QbDD) as a more efficient approach to product development. By combining physics-based models grounded in thermodynamics, material science, and fluid dynamics with data-driven models, QbDD enables proactive process modeling. This integration reduces the reliance on extensive empirical data, resulting in shorter development timelines and lower costs (Mustoe, 2025). Additionally, QbDD enhances process understanding and expands the flexibility of the design space (DS), leading to more robust and adaptable manufacturing processes. This paper compares different types of models, including empirical and hybrid, to assess the solubility and miscibility of ibuprofen in four polymer matrices. The goal is to shift from purely data-driven empirical models toward more physics-informed, mechanistic, and hybrid modeling approaches.

A range of models has been evaluated to predict the solubility and miscibility of active pharmaceutical ingredients (APIs) within polymer matrices in amorphous solid dispersions (ASDs). These models vary in complexity and theoretical basis, from purely empirical equations derived from experimental data to advanced physics-based models based in molecular theory. Common approaches include the Flory–Huggins theory for polymer–drug miscibility, group contribution methods like Fedors, van-Krevelen, and Just-Breitkreutz methods, and equations of state such as PC-SAFT for phase behavior prediction. Additionally, hybrid models, such as Gordon–Taylor and Kwei equations, are widely used to estimate glass transition temperatures (T_g_), which serve as indicators of miscibility and physical stability. The choice of model depends on the available data, the nature of the drug-polymer system, and the desired balance between accuracy and computational efficiency.

The use of Hildebrand solubility parameter is a concept used to estimate the cohesive energy density (CED**)** of a substance, which is a measure of the strength of intermolecular forces in that substance. However, it does not separate the different types of interactions (polar, hydrogen bonding, etc.), which may limit its ability to model more complex systems. So, the Hansen Solubility Parameter (HSP) is a more detailed and versatile approach than the Hildebrand method, especially in systems where interactions such as polarity and hydrogen bonding are significant.

Based on this principle, group contribution (GC) methods, such as those developed by Fedors (1974) and van Krevelen (1976), calculate solubility parameters by considering the additive contributions of functional groups. The HVK method, also known as the Hoftyzer-van Krevelen method, is a group contribution method used to estimate the Hildebrand solubility parameter (δ), which describes the cohesive energy density (CED) of a substance. The Just-Breitkreutz (JB) method extended the application of the GC methods specifically to the pharmaceutical field, emphasizing solid-state solubility determination rather than liquid-state (Just et al., 2013). This approach is widely used in literature to screen for different drugs and polymers (Kitak et al., 2015; Petříková et al., 2021). For this work, the Fedors, van-Krevelen, and Just-Breitkreutz methods were initially employed to screen a range of polymers with the potential to be a suitable matrix to solubilize ibuprofen. This approach enabled rapid, preliminary screening and served as a hybrid strategy that complemented and informed subsequent verification using thermodynamic properties to describe polymer-solvent phase behavior.

API-polymer solubility can be experimentally determined using differential scanning calorimetry-based methods by examining recrystallization and dissolution behaviors of APIs in polymer matrices (Mahieu et al., 2013; Mathers et al., 2021). How well an active pharmaceutical ingredient dissolves in a polymer is a crucial factor in designing stable amorphous solid drug formulations.

Nishi and Wang (1975) were the first to report melting point depression (MPD) in amorphous-crystalline polymer blends and to link it to miscibility. This phenomenon has since been investigated with poorly soluble APIs and amphiphilic polymers, showing temperature depressions that can vary based on API-polymer interactions, sample preparation, and DSC methodology (Weuts et al., 2004; Jijun et al., 2011; Iemtsev et al., 2020). The end-set evaluation of enthalpic peaks provides the most consistent data for melting analysis, though high polymer content can increase viscosity and thus incorrect MPD values. Based on Flory-Huggins (FH) theory, the obtained near-melting interaction parameter between drug and polymer (χ) can be extrapolated to lower temperatures and compositions, enabling the construction of Gibbs free energy of mixing and phase diagrams. While FH theory remains one of the most widely used models in the field of amorphous solid dispersion (ASD) development, it has several limitations. Originally developed to describe the phase behavior of polymers or polymer mixtures in solution, it may not fully capture the complexity of phase behavior in ASDs. Specifically, the simple FH interaction parameter (χ) alone may not accurately represent the interactions within these molecular mixtures, as it does not account for specific molecular forces such as hydrogen bonding, ionic interactions, or van der Waals forces, which play crucial roles in ASDs (Anderson, 2018a, b).

The glass transition temperature (T_g_) is the temperature at which an amorphous material transitions from a hard, glassy state to a more rubbery, flexible state which can be measure by differential scanning calorimetry (DSC). This transition is sensitive to the molecular interactions in the system. In a miscible system (where the drug and polymer are well-integrated at the molecular level), the T_g_ will typically shift compared to the individual components. A single T_g_ observed for ASD suggests that the drug and polymer are miscible and have formed a homogeneous mixture. If the T_g_ of the ASD is lower or higher than the T_g_ of the pure components (drug or polymer), that can provide insights into the strength and nature of interactions between the drug and polymer. For example, lower T_g_ in the ASD compared to the pure polymer may indicate good interaction between the drug and polymer, which helps to reduce rigidity and improve dissolution properties. A higher glass transition temperature (T_g_) may indicate that the drug is more tightly “locked” within the polymer matrix, potentially influencing its release profile. The physical stability of an amorphous solid dispersion (ASD) is closely related to its T_g_. A higher T_g_ generally suggests greater stability, as reduced molecular mobility lowers the risk of crystallization. In contrast, a lower T_g_ may imply increase susceptibility to phase separation or crystallization over time, which can compromise drug performance. A system T_g_ can be experimentally measured and theoretically calculated using various models and approaches depending on the system under study and the available data. For simpler systems, equations like Gordon-Taylor and Kwei models may suffice, while more complex systems might require simulation-based methods such as molecular dynamics. These methods are particularly useful when experimental data is difficult or impossible to obtain, as they offer predictions based on molecular-level interactions, free volume, and thermodynamic principles.

In recent years, the Perturbed-Chain Statistical Associating Fluid Theory (PC-SAFT) has gained attention as a more advanced model for API-polymer phase behavior modeling (Prudic et al., 2014; Lehmkemper et al., 2017b; Luebbert et al., 2017). PC-SAFT is a thermodynamic model that calculates Helmholtz energy by summing contributions from molecular repulsion, dispersion and association. Although PC-SAFT requires additional initial effort to define the complex molecular parameters, they more accurately represent chemical interactions and molecular size differences in API-polymer compositions.

Constructing a thermodynamic phase diagram is a valuable approach for analyzing the solubility and miscibility of an API in a polymer. This enables, for instance, the identification of critical formulation parameters for hot melt extrusion (HME) and can be used to predict stability through mathematical modeling. The solubility curve (solid-liquid equilibrium, SLE) represents the API's equilibrium solubility in the polymer and defines the maximum stable drug loading at a given temperature. Beyond the solubility limit, liquid-liquid phase separation (LLPS), also called amorphous-amorphous phase separation (AAPS), may occur, explained by the binodal and spinodal curves (Qian et al., 2021). The binodal curve delineates the phase boundary between a homogeneous single-phase region and a two-phase region, where one phase is drug-rich and the other is polymer-rich. The area between the binodal and spinodal curves represents the metastable region, where phase separation is thermodynamically favored but kinetically hindered. In this region, the system remains temporarily stable unless triggered by external factors such as temperature and humidity changes (Klueppelberg et al., 2023; Moseson et al., 2024). The spinodal curve defines the boundary beyond which spontaneous phase separation occurs.

In this work, ibuprofen (IBU) was selected as model API, a class II API according to the biopharmaceutical classification system (BCS) due to its low aqueous solubility and high permeability. Because of its inherent low melting temperature (T_m_) and glass-transition temperature (T_g_) resulting in high mobility and low configurational entropy, IBU is deemed class IV in the recently proposed amorphous classification system (ACS), representing a challenging API for amorphous–based formulations, (Zhou et al., 2019). Binary blends containing Kollidon® VA64, Kollidon® 17PF, HPMCAS, and Eudragit® EPO were systematically investigated to evaluate the solubility and miscibility of these systems using empirical and hybrid models.

## 2 Materials

**Fig. 1:**
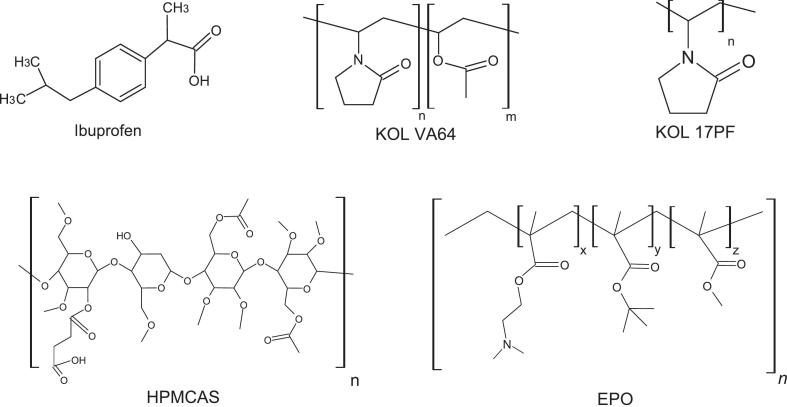
Ibuprofen (IBU), Kollidon® VA64 (KOL VA64), Kollidon® 17PF (KOL 17PF), HPMCAS and Eudragit EPO (EPO) molecular structures.

IBU, KOL VA64 (Mw 65 kDa) and KOL 17PF (Mw 11 kDa) were kindly supplied by BASF (Ludwigshafen, Germany). HPMCAS (AQOAT- AS-LMP) was donated by Harke Chemlink (UK) and EPO (Eudragit EPO®) by Evonik Pharma Polymers (Darmstadt, Germany). API and polymers physicochemical properties are represented on Table 1 and their chemical structure in Fig. 1. The true density (ρ), glass transition temperature (T_g_), melting transition temperature (T_m_)_,_ molar enthalpy of fusion (Δ_fus_H) and change in heat capacity (ΔC_p_) were determined experimentally.

**Table 1:** Ibuprofen and polymers true density and thermodynamic properties.

Compound	*M*_w_ (g mol^−1^)	ρ (g cm^−3^)	*T*_g_ (°C)	*T*_m_ (°C)	Δ_fus_H(kJ mol^−1^)	Δ*C*_*p*_ (J mol^−1^ K^−1^)
Ibuprofen	206.28	1.11	−44.68	75.08	24.68	70.91
KOL VA64	65,000	1.22	109.3	–	–	–
KOL 17PF	11,000	1.23	132	–	–	–
HPMCAS (AQOAT AS-LMP)	18,000	1.30	122.5	–	–	–
Eudragit EPO	47,000	1.14	57.2	–	–	–

## 3 Methods

### 3.1 Group contribution (GC) theory

The Fedors solubility parameter ($\delta_{t})$ is defined as per Eq. 1, where ${\mathrm{\Delta H}}_{v}$ corresponds to energy of vaporization, R is universal gas constant, T is the temperature and $V_{m}$ denotes the molar volume.

(1) $$\delta_{t}=\sqrt{\frac{{\mathrm{\Delta H}}_{v}-\mathrm{RT}}{V_{m}}}$$

The HVK and JB methods for solubility parameters are comprised of three contribution terms: dispersive force (or London force) ($F_{\mathrm{di}}$), polar force ($F_{\mathrm{pi}}$), and hydrogen-bonding energy ($E_{\mathrm{hi}}$), represented by equations below (Eq. 2 to Eq. 5):

(2) $$\delta_{d}=\frac{\sum_{i}F_{\mathrm{di}}}{\sum_{i}V_{i}}$$

(3) $$\begin{array}{c} \delta_{p}=\sqrt{\frac{\sum_{i}F_{\mathrm{pi}}^{2}}{\sum_{i}V_{i}}} \end{array}$$

(4) $$\begin{array}{c} \delta_{h}=\sqrt{\frac{\sum_{i}E_{\mathrm{hi}}}{\sum_{i}V_{i}}} \end{array}$$

(5) $$\begin{array}{c} \delta_{t}=\sqrt{\delta_{d}^{2}+\delta_{p}^{2}+\delta_{h}^{2}} \end{array}$$

The API-polymer miscibility can be estimated by their distance ($R_{a\left(v\right)})\;$on a Bagley Plot, a two-dimensional projection obtained by combining the volume–dependent solubility parameter ($\delta_{v})$ as a function of $\delta_{h}$ (Eq. 6 and Eq. 7).

(6) $$\begin{array}{c} \delta_{v}=\sqrt{\delta_{d}^{2}+\delta_{p}^{2}} \end{array}$$

(7) $$\begin{array}{c} \;R_{a\left(v\right)}=\sqrt{4{\left(\delta_{\mathrm{vpolymer}}-\delta_{\mathrm{vAPI}}\right)}^{2}+{\left(\delta_{\mathrm{hpolymer}}-\delta_{\mathrm{hAPI}}\right)}^{2}} \end{array}$$

According to the thermodynamic solution theory, the relationship between the Flory–Huggins' interaction parameter (χ) and the solubility parameters (δ) is given by eq. (8) where $V_{0}$ is the lattice site volume, R is the universal gas constant and T is the temperature (Eq. 8).

(8) $$\begin{array}{c} \chi =\frac{V_{0}}{\mathrm{RT}\;}{\left(\delta_{\mathrm{API}}-\delta_{\mathrm{polymer}}\right)}^{2} \end{array}$$

### 3.2 Flory-Huggins (FH) theory

To extrapolate and predict IBU solubility across lower temperatures, the API-polymer end-set temperature was modeled using the FH model (Eq. 9). Here, $T_{m}$ represents the end-set melting temperature of the pure IBU in kelvin, and $\Delta_{\mathrm{fus}}H$ denotes its molar enthalpy of fusion. R is the universal gas constant, while $\phi_{\mathrm{API}}$and $\phi_{\mathrm{polymer}}$ correspond to the volume fractions of IBU and polymer, respectively. The API–polymer volume ratio (m) is the molar volume ratio of the polymer to the reference lattice site occupied by the API, which can be calculated as the ratio between molecular volumes (*N*_*A*_/*N*_*B*_), considering components' molecular weight ($M_{w})$ and true density (ρ) which were measured using a helium pycnometer (Micromeritics, US), with a 3.5 cm^3^ sample holder submitted to a 10-cycle method (Eq.10). The term χ is the Flory–Huggin's interaction parameter accounting for API–polymer interactions. Because it is affected by compositional and temperature effects this relationship is frequently simplified as the entropic contribution is obtained by the constant A, while the temperature-dependent enthalpic contribution is represented by B (Eq.11) (Tian et al., 2018a, b).

(9) $$\begin{array}{c} \frac{1}{T}-\frac{1}{T_{m}}=-\frac{R}{\Delta_{\mathrm{fus}}H}\;\left[{\mathrm{ln\phi}}_{\mathrm{API}}+\left(1-\frac{1}{m}\right)\phi_{\mathrm{polymer}}+\chi \phi_{\mathrm{polymer}}^{2}\right] \end{array}$$

(10) $$\begin{array}{c} m=\frac{N_{B}}{N_{A}}=\frac{\frac{M_{w\left(\mathrm{polymer}\right)}}{\rho_{\mathrm{polymer}}}}{\frac{M_{w\left(\mathrm{API}\right)}}{\rho_{\mathrm{API}}}} \end{array}$$

(11) $$\begin{array}{c} \chi =A+\frac{B}{T} \end{array}$$

In a binary system comprising a polymer and a low molecular weight drug, the Gibbs free energy of mixing as described by the Flory–Huggins model consists of a combinatorial component and an interaction component, resulting in Eq.12 (Zhao et al., 2011). Considering that the phase boundary is defined by the common tangent of free energy, the metastable and unstable regions can be calculated numerically solving by $\Phi_{\mathrm{API}}$ and setting the first and second derivatives to 0 (Eq.13 and Eq. 14).

(12) $$\begin{array}{c} {∆}_{\mathrm{mix}}G=\mathrm{RT}\left(\Phi_{\mathrm{API}}+\ln\Phi_{\mathrm{API}}+\frac{\Phi_{\mathrm{polymer}}}{m}\ln\Phi_{\mathrm{polymer}}+\chi \Phi_{\mathrm{API}}\Phi_{\mathrm{polymer}}\right) \end{array}$$

(13) $$\begin{array}{c} \left(\frac{\delta }{\delta {ɸ}_{\mathrm{API}}}\left(\frac{{∆}_{\mathrm{mix}}G}{\mathrm{RT}}\right)\right)=\ln{ɸ}_{\mathrm{API}}+1-\left(\frac{1}{m_{\mathrm{polymer}}}\right)-\left(\frac{1}{m_{\mathrm{polymer}}}\right)\ln\left(1-{ɸ}_{\mathrm{API}}\right)+\left(1-2{ɸ}_{\mathrm{API}}\right)\chi =0 \end{array}$$

(14) $$\begin{array}{c} \left(\frac{\delta^{2}}{\delta {ɸ}_{\mathrm{API}}^{2}}\left(\frac{{∆}_{\mathrm{mix}}G}{\mathrm{RT}}\right)\right)=\frac{1}{{ɸ}_{\mathrm{API}}}+\frac{1}{m_{\mathrm{polymer}}{ɸ}_{\mathrm{polymer}}}-2\chi =0 \end{array}$$

### 3.3 Perturbed-chain statistical associating fluid (PC-SAFT) theory

PC-SAFT is a thermodynamic model to calculate the residual Helmholtz energy $\left(a^{\mathrm{res}}\right)$ by summing up different contributions caused by molecular repulsion (a^hc^), attraction (dispersion a^disp^) and association (a^assoc^) (Eq. 15) (Gross and Sadowski, 2001).

(15) $$\begin{array}{c} a^{\mathrm{res}}=a^{\mathrm{hc}}+a^{\mathrm{disp}}+a^{\mathrm{assoc}} \end{array}$$

The $a^{\mathrm{res}}$ is used to calculate the compressibility factor (Z) and consequently the fugacity coefficient (φ) (Eq. 16 and Eq.17). An $\gamma_{i}$ is obtained with explicit contributions from molecular interactions, including size, shape, dispersion forces, and association effects, through its segment-based equation of state (Eq.18)

(16) $$\begin{array}{c} Z=1+\rho \left[\partial \left(\frac{a^{\mathrm{res}}/k_{b}\;T}{\partial \rho }\right)\right] \end{array}$$

(17) $$\begin{array}{c} \ln\varphi_{i}^{L}=\frac{\mu_{i}^{\mathrm{res}}}{K_{B}T}-\ln Z \end{array}$$

(18) $$\begin{array}{c} \gamma_{i}=\frac{\varphi_{i}^{L}}{\varphi_{0,i}^{L}} \end{array}$$

The mole fraction API solubility in a liquid amorphous phase is obtained by Eq.19, $\gamma_{\mathrm{API}}$ is the activity coefficient of the API in the mixture, $\Delta_{\mathrm{fus}}H$ is the API molar enthalpy of fusion, $\Delta_{\mathrm{fus}}C_{p}$ is the difference between pure API molar isobaric liquid and solid heat capacity.

(19) $$\begin{array}{c} x_{\mathrm{API}}=\frac{1}{\gamma_{\mathrm{API}}}\exp\left[-\frac{\Delta_{\mathrm{fus}}H}{\mathrm{RT}}\left(1-\frac{T}{T_{m}}\right)-\frac{\Delta_{\mathrm{fus}}C_{p}}{R}\left(1-\frac{T_{m}}{T}+\ln\frac{T_{m}}{T}\right)\right] \end{array}$$

A binary interaction parameter $k_{\mathrm{ij}}$ (Eq. 20) is used to correct for deviations the pure-component energy parameters and is the only parameter fitted to experimental binary data when using PC-SAFT (Christian Marc, 2018)

(20) $$\begin{array}{c} k_{\mathrm{ij}}=k_{\mathrm{ij},\mathrm{int}}+k_{\mathrm{ij},\mathrm{slope}}\;T\left[K\right] \end{array})\;$$

Upon AAPS, two coexisting phases (API–poor and API–rich phases, defined as L^1^ and L^2^ respectively) with distinct molar-fraction compositions x are defined as per Eq. 21 and Eq.22. The equilibrium composition of two existing amorphous phases can be determined by simultaneously resolving equations at different temperatures of interest (Luebbert et al., 2017).

(21) $$\begin{array}{c} x_{\mathrm{API}}^{L1}\gamma_{\mathrm{API}}^{L1}=x_{\mathrm{API}}^{L2}\gamma_{\mathrm{API}}^{L2} \end{array}$$

(22) $$\begin{array}{c} x_{\mathrm{polymer}}^{L1}\gamma_{\mathrm{polymer}}^{L1}=x_{\mathrm{polymer}}^{L2}\gamma_{\mathrm{polymer}}^{L2} \end{array}$$

In essence, for an associating compound the use of six parameters is required to account for its interactions, which are: the number of segments (m_i_), segment diameter (σ_i_), the dispersion energy parameter (ε_i_/k), association energy parameter (ε_iassoc/k_), association volume (ki_assoc_) and number of associations (donors/acceptors – N_iassoc_) (Table 2).

**Table 2:** API and polymers PC-SAFT parameters.

Compound	*m*_i_	σ_i_ (Å)	ε_i_/*k* (K)	ε_i_^assoc^/*k* (K)	*k*_i_^assoc^	*N*_i_^assoc^
Ibuprofen ^a^	2.52	4.43	374.65	879.42	0.03	2.00
KOL VA64 ^b^	2420.99	2.95	205.27	0.00	0.02	653.00
KOL 17PF ^c^	407.00	2.71	205.60	0.00	0.02	89.88
HPMCAS (AQOAT AS-LMP) ^d^	895.70	2.91	316.87	2454.87	0.02	111.00
Eudragit EPO ^e^	1645.00	3.74	258.89	0.00	0.02	848.00

Reference data: a – (Ruether and Sadowski, 2009), b – (Luebbert et al., 2017), c – (Prudic et al., 2014), d – (Lehmkemper et al., 2017a), e – Amofor® GmbH

### 3.4 Kyeremateng empirical equation

An empirical approach (Eq. 23) to determine the API–polymer solubility curve was developed by Kyeremateng (Kyeremateng et al., 2014) using DSC measurements. In this method, Tₛ represents the API solubility temperature, Tₘ is the melting point of the pure API, x is the API content in the mixture, and A, b, and C are fitting parameters. In their original study various API–polymer combinations were evaluated finding b values close to −0.05, when C was set to zero. In an alternative approach explored by Mathers et al., 2021, A and b are first determined with C fixed at zero, followed by C optimization. In this work, we have compared both approaches, referred to here as ‘One-step fitting’ and ‘Two-steps fitting’, respectively.

(23) $$\begin{array}{c} T_{s}=-Ae^{\mathrm{bx}}+T_{m}+C \end{array}$$

### 3.5 Gordon-Taylor and Kwei theories

The glass transition temperatures of IBU-KOL VA64, IBU-KOL 17PF, IBU-HPMCAS, IBU–EPO blends were modeled using the Gordon-Taylor (GT) and Kwei equations, as a function of API content (% *w*/w) (Eq. 24).

(24) $$\begin{array}{c} T_{g}=\frac{w_{\mathrm{API}}T_{g,\mathrm{API}}+K_{\mathrm{GT}}w_{\mathrm{polymer}}T_{g,\mathrm{polymer}}}{w_{\mathrm{API}}+K_{\mathrm{GT}}w_{\mathrm{polymer}}} \end{array}$$

The constant $K_{\mathrm{GT}}$ was both calculated using the Simha-Boyer rule (Eq. 25) and fitted experimental data.

(25) $$\begin{array}{c} K_{\mathrm{GT}}=\frac{\rho_{\mathrm{API}}\;{T_{g}}_{\mathrm{API}}}{\rho_{\mathrm{polymer}}\;{T_{g}}_{\mathrm{polymer}}} \end{array}$$

Kwei investigated polymer mixtures and proposed an empirical modeling equation, where *k* ($\text{similar to}\;K_{\mathrm{GT}})$ and *q* are fitted parameters (Eq. 26), based on intermolecular interactions between the mixture components, particularly hydrogen–bonding (Kwei, 1984).

(26) $$\begin{array}{c} \;T_{g}=\frac{w_{\mathrm{API}}T_{g,\mathrm{API}}+K_{\mathrm{GT}}w_{\mathrm{polymer}}T_{g,\mathrm{polymer}}}{w_{\mathrm{API}}+K_{\mathrm{GT}}w_{\mathrm{polymer}}}+q\;w_{\mathrm{API}}\;w_{\mathrm{polymer}} \end{array}$$

### 3.6 Model accuracy evaluation

Model accuracy evaluation was performed through average absolute relative deviation (AARD) and absolute relative deviation (ARD), as per equations below (Eq. 27 and Eq. 28).

(27) $$\begin{array}{c} \mathrm{AARD}=\frac{1}{N}\;\sum_{i=1}^{N}\left|\frac{T_{\mathrm{measured}}-T_{\mathrm{predicted}}}{T_{\mathrm{measured}}}\right|\;X\;100 \end{array}$$

(28) $$\begin{array}{c} \mathrm{ARD}=\frac{1}{N}\;\sum_{i=1}^{N}\left(\frac{T_{\mathrm{measured}}-T_{\mathrm{predicted}}}{T_{\mathrm{measured}}}\right)\;X\;100 \end{array}$$

### 3.7 Preparation of API–polymer physical mixture

API–polymers mixtures studied (95–30 % w/w) were obtained by precisely weighing and pestle and mortar mixing for 10 min, to avoid unwanted amorphization. Geometric mixing was performed to ensure API homogeneous distribution within the samples.

### 3.8 Melting point depression (MPD)

Differential scanning calorimetry measurements were performed in triplicate with a DSC TA25 (Waters, USA), refrigerated using a RCS90 cooling system, to determine T_m_ and T_g_ from crystalline IBU and investigated blends (95–30 w/w%). During measurements, the equipment was purged at the rate of 50 mL/min (nitrogen). Samples were prepared by pestle and mortar mixing for approximately 10 min and submitted to a heat-quench-heat cycle. In the first heating cycle samples were heated from 0 to 130 °C, at varying rates (1 or 5 °C/min), followed by quenching (10 °C/min) to −90 °C, and a second heating cycle of 0 to 130 °C (1 or 5 °C/min) for the measurement of the T_g_. Samples' melting enthalpy variation, as a function of composition, were measured using TRIOS software. Melting depression data was modeled using FH, Kyemerateng empirical equation (MATLAB® - vR2024b) and PC-SAFT (SOLCALC software – Amofor®). The T_g_ values were measured using the second heating cycle.

## 4 Results and discussion

### 4.1 Group contribution modeling

**Fig. 2:**
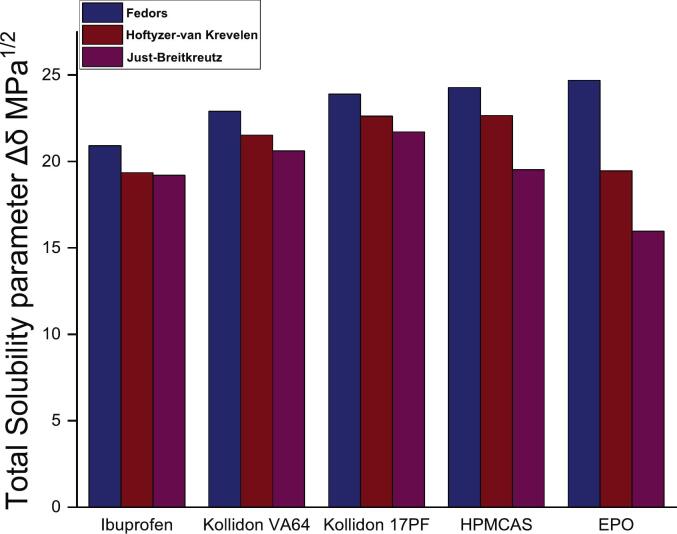
Total solubility parameter Δδ_t_ obtained using Fedors, Hoftyzer van-Krevelen and Just-Breitkreutz methods.

The use of group contribution method allows for rapid screening across a wide range of formulations, facilitating the selection of suitable API-polymer combinations with favorable solubility parameter values. This is particularly useful during early product development, in which limited material might be available. Fig. 2 illustrates the total solubility (δ_t_) variation for all compounds across different GC methods. A common observed trend is that Fedors-derived, which are obtained only using a single contribution (Eq. 1) outputs, are higher than its counterparts. Differences become more significant when compared to JB method, particularly for HPMCAS (Fedors vs JB Δδ_t_ = 4.78 MPa^1/2^) and EPO (Fedors vs JB, Δδ_t_ = 8.72 MPa^1/2^). This finding is well correlated with an already reported trend (Kitak et al., 2015) explained by lower contribution figures from JB, probably due to its difference in calculation by using solid-state. According to Greenhalgh et al. (1999), a common rule of thumb suggests that API-polymer blends with Δδ_t_ < 7.0 MPa^1^/^2^ are likely to be soluble, while values exceeding 10 MPa^1^/^2^ tend to indicate phase separation. Such threshold is supported by blends of Itraconazole, Indomethacin, Griseofulvin, and Bisacodyl with various polymers, where Δδ_t_ values below 7.0 MPa^1/2^ were obtained, and solubility parameter was empirically confirmed. These systems presented a single T_g_ value in DSC data indicating a homogenous one phase systems (Sarode et al., 2013; Lee et al., 2023). The largest value observed in this study is −5.49 MPa^1/2^ for IBU–EPO blend, indicating that all API-polymer combinations tested should be considered soluble by this rule of thumb (Fig. 2).

**Fig. 3:**
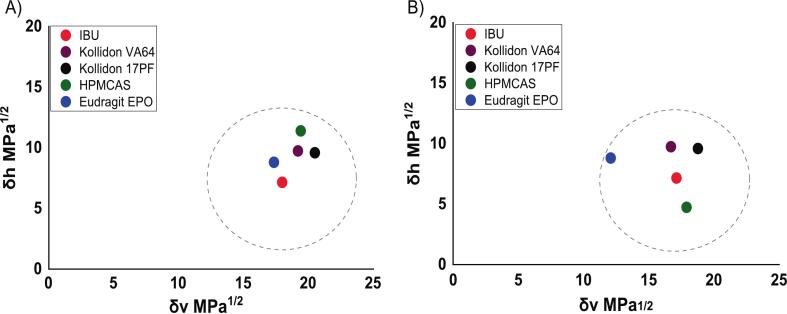
Bagley plots depicting R_av_ parameter obtained using a) HVK and b) JB methods.

While Δδ_t_ is useful for initial solubility screening, it provides good approximations only if the weak interactions are predominant in the blend (Zhu et al., 2021). Bagley plots, on the other hand, offer a more detailed approach by presenting solubility parameters in two-dimensional space, typically plotting dispersive and polar components as a single component and allowing a clearer visualization of the distinct interaction forces influence within each API-polymer blend. Recent studies have highlighted the utility of a two-dimensional Bagley's plot (R_a(v)_) for visualizing API–polymer solubility parameters systems with diverse structural features (Fig. 3) (Choudhury et al., 2023; Kolisnyk et al., 2023). The R_a(v)_ values exhibit a degree of variability between the different calculation methods, primarily due to the distinct group contribution values from each database (Fig. 2). Nonetheless, the overall trend indicates that PVP–based polymers (KOL VA64 and KOL 17PF) are likely to be better solvents for IBU than HPMCAS, to which values almost fall out of solubility range. IBU-EPO calculated using JB method (R_a(v)_ = 5.34 MPa^1^/^2^) suggests a less soluble system, contrasting with the HVK derived result (R_av_ = 1.78 MPa^1^/^2^). This discrepancy arises from differences in the dispersive energy ($\delta_{d}$) component between the two methods, with JB yielding 12.09 MPa^1^/^2^ and HVK yielding 17.35 MPa^1^/^2^.

Eq. 8 shows the FH interaction parameter (χ), a useful comparison parameter which accounts the molecular lattice volume. In general, there is an inverse relationship between (χ) and miscibility, with negative values indicating better compatibility between molecules. The poorest miscibility (χ =1.13 MPa^1/2^) was obtained for the IBU–EPO system using Fedors' method, which is based on the Hildebrand solubility parameter and does not account for specific interactions. This result contrasts with evidence of strong hydrogen bonding in this system, as reported elsewhere (Yani et al., 2017). This interaction was probably better captured by HVK, yielding the lowest (χ) (0 MPa^1/2^). Significant variability was also found for HPMCAS using the 3 different methods. These discrepancies highlight the limitations of these methodologies, as each identified a different polymer as the most compatible candidate. Such variability can impair the screening phase, underlining the need for a more comprehensive evaluation framework. More detailed of the solubility and interaction parameter values obtained from CG methods can be found in Table S1.

### 4.2 Flory-Huggins modeling

**Fig. 4:**
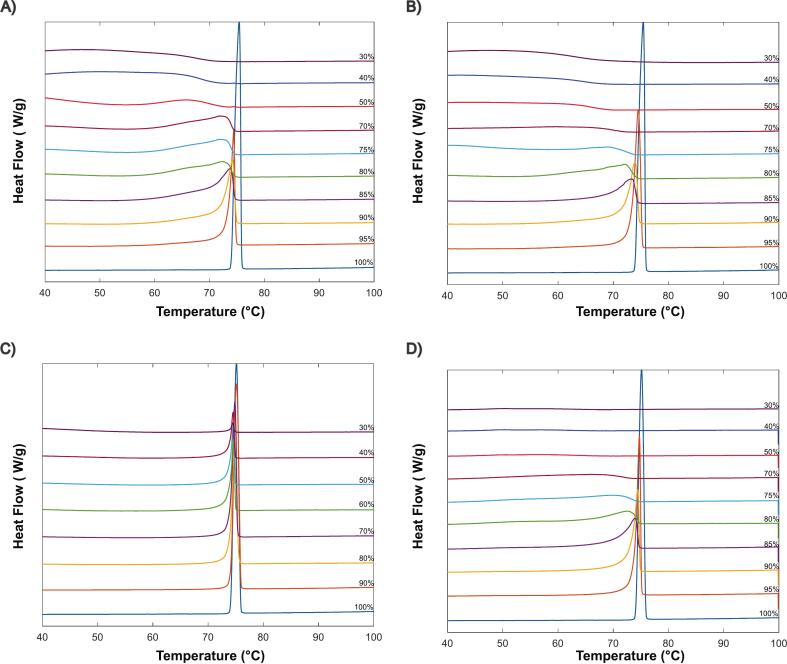
DSC data at different API-polymer compositions, showing melting point depression (1 °C/min). A) KOL VA64. B) KOL 17PF. C) HPMCAS. D) EPO.

**Fig. 5:**
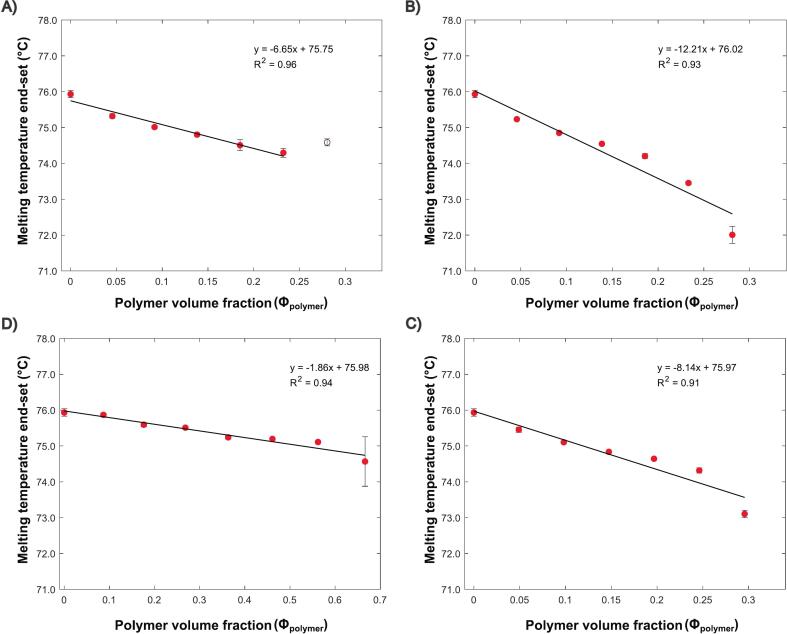
Linear fitting from MPD data for the different compositions evaluated for (A) KOL VA64, (B) KOL 17PF, (C) HPMCAS and (D) EPO.

A low heating rate of 1 °C/min was utilized for the API/polymer thermal behavior (Fig. 4**)**, since this approach yields values closer to true equilibrium (Mathers et al., 2023). KOL 17PF, showed more noticeable melting point depression in comparison to KOL VA64, probably explained by a combination of two factors: (1) stronger chemical interactions, shown by molecular dynamics simulation (Xiang and Anderson, 2019) and (2) lower molecular weight (11 kDa vs. 57 kDa), which facilitates chain mobility and interaction. At 70 %, IBU-KOL VA64 exhibits a plateau in melting depression, potentially from increased viscosity, thus this data point was excluded from the linear fitting (Fig. 5). Minimal depression was seen in HPMCAS blends with low polymer content (0–30 %), indicating weak interactions, corroborating with previous findings (Iemtsev et al., 2020). Blends with higher polymer content (50–70 %) still have crystalline drug peaks observable, indicating limited solubility, contrasting to KOL VA64 and KOL17PF. A clear linear depression trend for IBU–EPO (Fig. 5) reflects blend solubility, consistent with prior studies (Yang et al., 2019). More detailed information about melting temperature values and 5 °C/min experiments can be seen in Tables S2-S5 and Figs. S1-S3.

#### 4.2.1 Interaction parameter

**Fig. 6:**
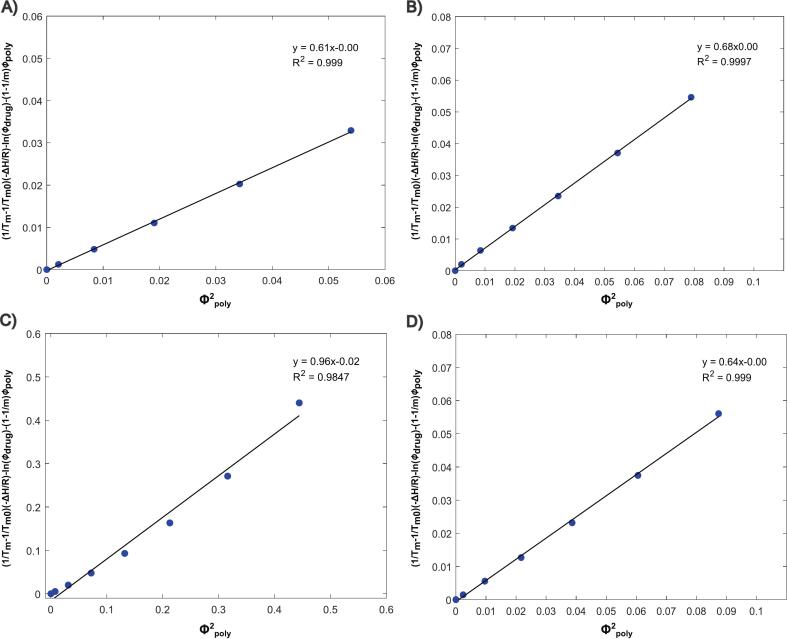
High temperature interaction parameter (χ) plots for the compositions tested (A) KOL VA64, (B) KOL 17PF, (C) HPMCAS and (D) EPO.

A single χ can be deconvoluted from MPD data's slope rearranging Eq. 9 as a function of $\phi_{\mathrm{polymer}}^{2}$ providing a more straightforward comparison between the 4 polymers investigated. As shown in Fig. 6**,** every blend tested yielded positive χ values around 0.65, while IBU–HPMCAS exhibited the highest value (0.96). These results are in agreement with other works reported in literature (Marsac et al., 2009; Deshmukh, 2014; Maniruzzaman et al., 2015). In general, a negative χ indicates that the attraction between the polymer-drug pair is stronger than the average attraction between polymer–polymer and API–API pairs. This ultimately means that drug molecules are more likely to interact with polymer segments than with other API molecules in that environment. Conversely, positive values suggest that drug molecules and polymer segments are prone to interact with their own molecules (Marsac et al., 2009; Lin and Huang, 2010).

**Fig. 7:**
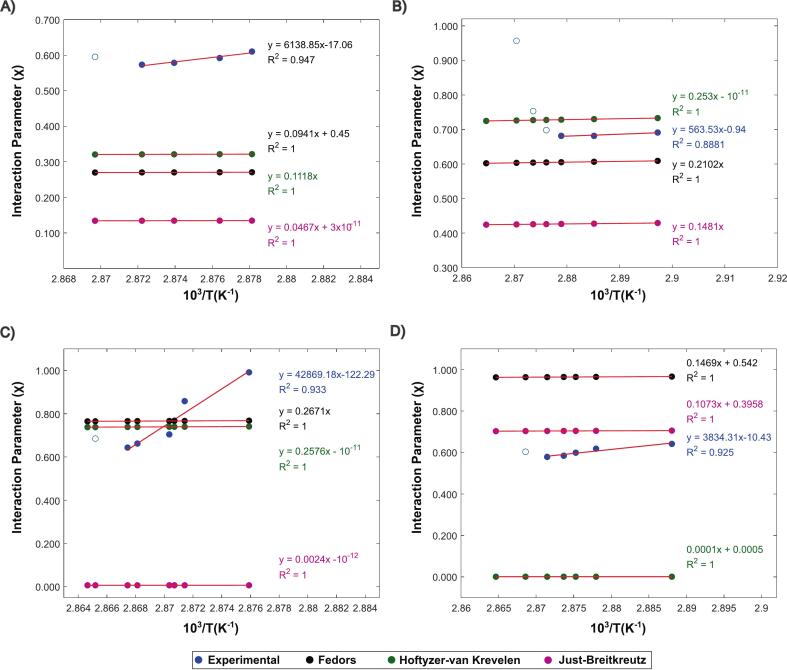
Interaction parameter calculated using experimental data and GC as a function of the inverted melting temperature in K (10^3^) for (A) KOL VA64, (B) KOL 17PF, (C) HPMCAS and (D) EPO. The solid line represents the best linear fitting for the data.

By measuring the melting point depression of mixtures with different drug-polymer ratios, a range of χ values can be derived at various temperatures. A linear correlation between χ and 1/T was expected across all compositions, as observed elsewhere in literature (Deshmukh, 2014). However, at higher drug loadings, particularly for KOL 17PF, a significant deviation from a linear trend can be seen (Fig. 7). Similar deviation trends were observed in blends containing indomethacin (IND), felodipine (FDP), itraconazole (ITZ), and, to a lesser extent, dapsone (DAP), when tested with different polymers (Zhao et al., 2011; Tian et al., 2013; Potter et al., 2018; Choudhury et al., 2023). As argued by Anderson (2018a), χ can display a nontrivial dependence on temperature and volume fraction of the polymer. This aligns with the idea that KOL 17PF, due to its lower M_w_, shows significant compositional effects. The same phenomenon was observed when IND–PVPK12 blends were evaluated (Mathers et al., 2023). For every blend, an upper critical solution temperature (UCST) behavior was found (A < 0, B > 0 – Table S9), generally the most common system type for drug-polymers submitted to this methodology (Tian et al., 2018b). The deviation from linearity is seen only at higher drug loadings, therefore these data points were disregarded during fitting, allowing a more robust extrapolation.

Using Eq. 8, a set of χ values from GC methods can be obtained at the melting temperatures of the compositions, allowing for a comparison between in-silico predictions and experimental data. In general, a significant discrepancy exists between experimental χ values and those derived from GC methods, with only a few overlapping points observed from Fedors and HVK. Moreover, the distinct slope present in the experimental data is absent in the theoretical results, indicating that the GC approach is incapable of accounting for compositional effects.

#### 4.2.2 Gibbs free energy of mixture

**Fig. 8:**
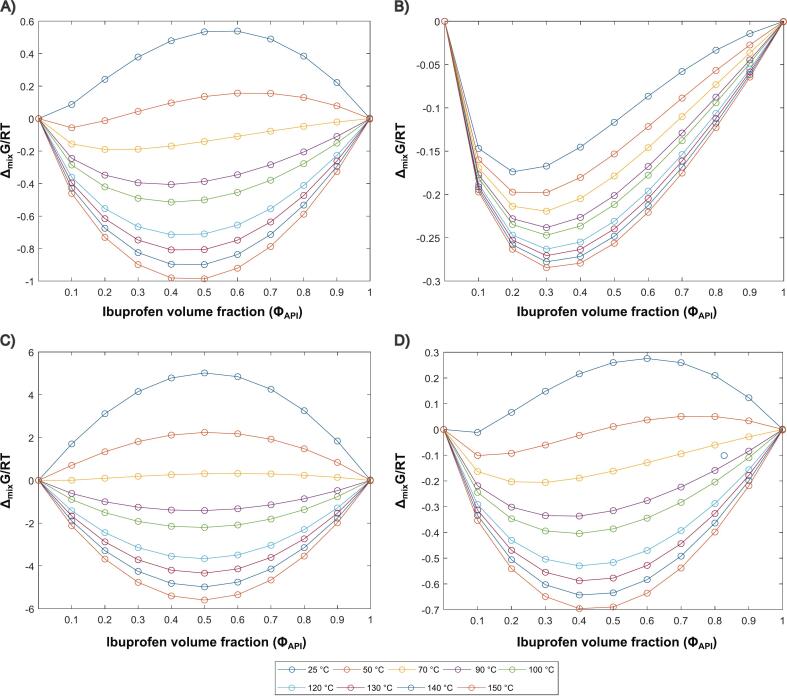
Δ_mix_G/RT as a function IBU volume fraction in different polymers: (A) KOL VA64, (B) KOL 17PF, (C) HPMCAS and (D) EPO at temperatures between 25 °C and 150 °C.

Fig. 8 illustrates the ∆_mix_G curves as a function of API-polymer composition and temperatures. It is clear that for temperatures higher than 80 °C all systems have negative ∆_mix_G. which normally indicates a single-phase system. However, phase separation may still occur if the composition exceeds the limits defined by the binodal curve. This curve, representing the miscibility boundaries, is derived using the common tangent construction on the ∆_mix_G curve and defines the composition range within which the mixture remains homogeneous. Beyond these limits, phase separation can take place even when ∆Gₘᵢₓ remains negative. It is also crucial to recognize that amorphous solid dispersions (ASDs) are complex systems where kinetic factors, such as molecular mobility and relaxation play a significant role. Therefore, to better predict potential instability, it is necessary to consider the glass transition temperature (T_g_) of the blends as well (Shekunov, 2020).

### 4.3 Kyemerateng empirical equation modeling

**Fig. 9:**
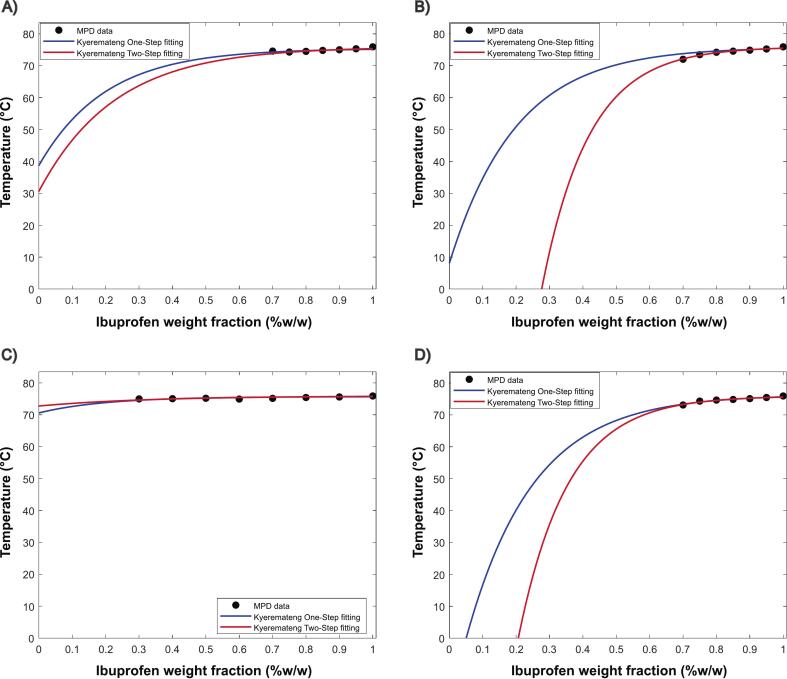
SLE curves extrapolated from MPD data using Kyemerateng empirical equation (One-Step and Two-Steps fitting). (A) KOL VA64, (B) KOL 17PF, (C) HPMCAS and (D) EPO.

Unlike the Fox model, which assumes ideal mixing, the Kyeremateng equation includes a correction factor that captures the depression or elevation of T_m_ due to molecular interactions. Fig. 9 shows that the degree of extrapolation varies between polymers: KOL17PF exhibits a steeper profile, while HPMCAS displays an almost flat trend. This behavior is associated with the extent of melting point depression induced by each polymer. The ‘One-Step fitting’ extrapolation approach employs a fixed ‘b’ value of −0.05, as reported by Kyeremateng et al. (2014) based on their evaluation of ten API–polymer blends. This value is, in principle, considered a reasonable approximation for other binary systems by the authors. In contrast, the ‘Two-Step fitting’ method imposes no constraint on the ‘b’ value and allows the ‘C' parameter to assume any value. The original authors suggested that the magnitude of ‘C' could reflect the strength of molecular interactions, similarly to the FH's χ, potentially accelerating polymer screening and formulation development. Looking at Table S6 when ‘Two-steps fitting’ is applied the ‘C' values for most depressed melting points data (17PF and EPO) is negative, whereas for the least depressed (KOLVA64 and HPMCAS) is positive, thus could be argued that such correlation is present, however not explicit. We prefer to interpret ‘C' primarily as a fitting parameter influenced by the optimization algorithm, lacking a clear physicochemical representation (Mathers et al., 2021). As expected, predictions of thermodynamic stability, particularly at lower temperatures, are highly sensitive to the fitting method used, as well as the experimental data range, which has also been observed in other IBU-based amorphous systems (Iemtsev et al., 2020). The Two-Step fitting presented slightly improve AARD values (Table S7) to MPD data and therefore we selected this approach to be compared with the other SLE curves later in this work.

### 4.4 PC-SAFT modeling

**Fig. 10:**
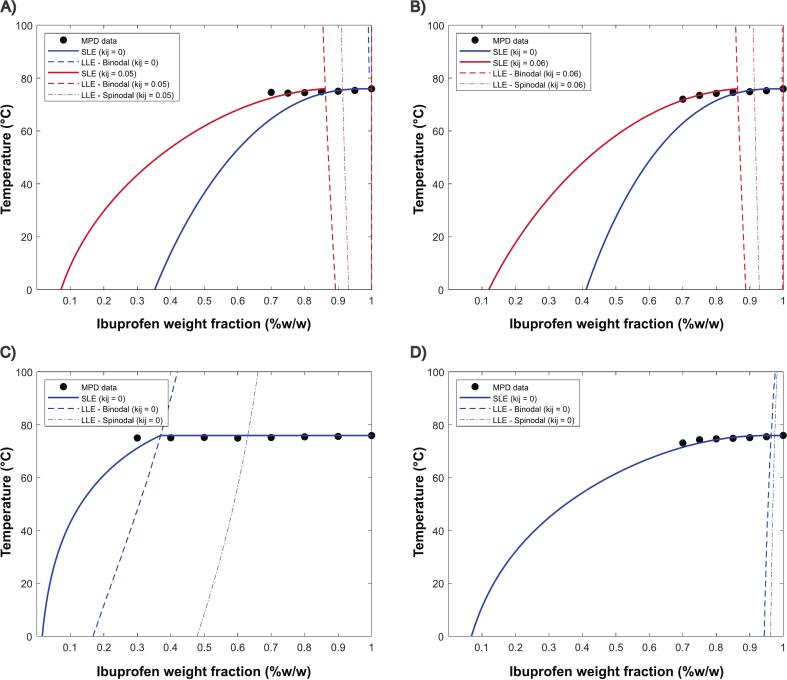
SLE curves extrapolated from MPD PC-SAFT (k_ij_ = 0 and k_ij_ ≠ 0) for (A) KOL VA64, (B) KOL 17PF, (C) HPMCAS and (D) EPO.

A comprehensive study conducted by Pavliš et al. (2023) using different sources of experimental data and comparing with PC-SAFT pure predictions showed good ranking correlation when comparing IBU and different polymers. Quantitative discrepancies could be attributed from the use of distinct methodologies, namely MPD and stepwise dissolution, conducted using different method parametrization, sample preparation and concentrations. Conversely, high accuracy between experimental data and PC-SAFT predictions for IBU was found by Christian Marc (2018). In fact, the predictions were also accurate to evaluate long-term stability under accelerated conditions. Initially, we were interested whether PC-SAFT pure predictions (k_ij_ = 0) are sufficiently accurate to predict API–polymer solubility, leveraging from its physics-based modeling. Considering IBU-KOLVA64 and IBU-KOL17PF, significant deviation between experimental values is found in pure predictions (k_ij_ = 0), with increasing blend polymer content (Fig. 10). Predictions made with all experimental data with positive fitted k_ij_ (k_ij_ = 0.05 and 0.06 respectively) improved model accuracy (AARD (%) = 1.50 and 0.86, respectively – from Table S7) and predicted AAPS zones at high API loadings (> 85 %*w*/w). For the other blends an improved prediction (k_ij_ = 0) is found possibly for distinct reasons. For the IBU–HPMCAS system, negligible melting point depression was observed, suggesting weak drug–polymer interactions. The model predictions deviated significantly only at high polymer content (70 % w/w), where such interactions may become more relevant. The presence of large liquid–liquid equilibrium (LLE) regions in the phase diagram supports this claim, indicating limited miscibility between the components. For IBU-EPO, from an experimental perspective the polymer's low T_g_ (∼42 °C) enables faster API–polymer equilibrium at the experimental heating rate (1 °C/min), as opposed to the remaining polymers (> 100 °C). To compare SLE and LLE curves we have selected those with the best fit to experimental data (fitted KOLVA64/KOL17PF, and pure predictions for HPMCAS and EPO) considering AARD (%) (Table S7).

### 4.5 Gordon-Taylor and Kwei modeling

**Fig. 11:**
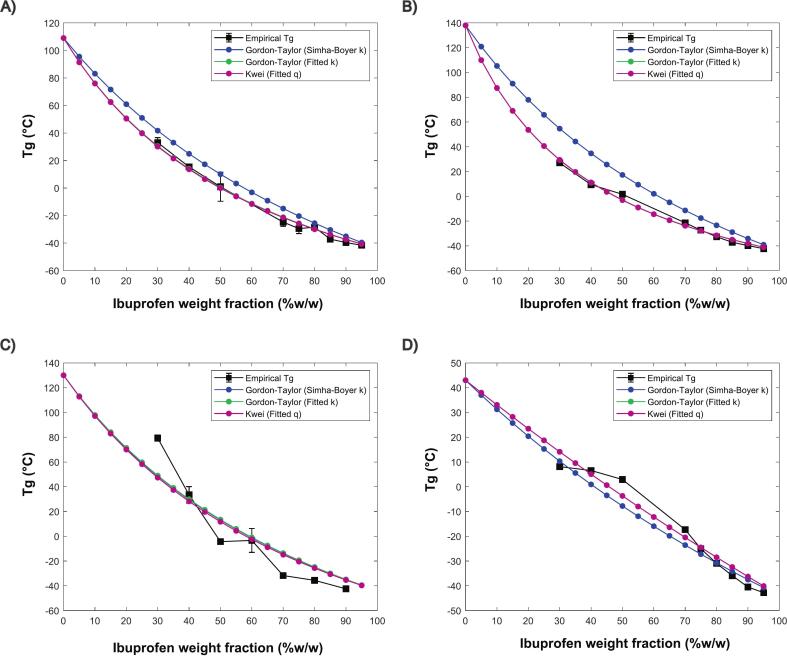
T_g_ modeling using GT (Simha-Boyer rule), GT (fitted k) and Kwei (fitted q) equations for IBU and (A) KOL VA64, (B) KOL 17PF, (C) HPMCAS and (D) EPO.

Temperature plays a major role on molecular mobility and ultimately on pharmaceutical formulation stability profile. It is frequently mentioned that ASD formulations kinetic instabilities are minimized when stored at T_g_ - 50 °C (Pandi et al., 2020), although it might be considered an oversimplified rule, as there is a complex interplay between formulation components and external conditions that affect stability. An inverse relationship between drug loading and T_g_ values is observed (Fig. 11), attributed to the plasticizing effect of IBU, which has a low T_g_ of −44.2 °C. This effect becomes more pronounced at higher drug loadings. Furthermore, T_g_ profile offers essential insights into processability, as samples with lower values of T_g_ face significant challenges during downstream extrusion, including reduced mechanical stability and an increased tendency for stickiness and deformation (Bhujbal et al., 2021). This indicates that blends exceeding 50 % w/w would be difficult to process, regardless of the polymer used.

Contradicting our initial thoughts, there is only a minor difference of T_g_ values from IBU-KOL VA64 and IBU-KOL 17PF mixtures, despite their distinct difference in T_g_ values of 109 °C and 132 °C, respectively. This indicates that IBU plasticization is more pronounced in KOL 17PF, likely due to stronger chemical interactions, agreeing with spontaneous solid–state amorphization found between IBU and PVP (Sekizaki et al., 1995).

Furthermore, deviations from prediction models can provide valuable insights about API–polymer intermolecular interaction likelihood (Baghel et al., 2016). KOL VA64 and KOL 17PF blends showed negative deviations from GT curves using a theoretical ‘k’ value, however, when ‘k’ and ‘q’-fitted values were used, there is satisfactory agreement with empirical values (Fig. 11) revealing that chemical interactions are occurring between the components. IBU–EPO has depicted drug loading-dependent variations, resulting on a ‘S-shaped’ T_g_ curve, as already observed for IND-PVPK12 (Mathers et al., 2021). Conversely, IBU-HPMCAS blend had poor correlation with every model tested, supporting chemical incompatibility. A more objective comparison between models (Table S8) shows that GT using Simha-Boyer approximation had higher ARD and AARD values, when compared to fitted GT and Kwei. The latter models have demonstrated similar performance showing minor deviations from experimental data, as already reported in elsewhere (Krummnow et al., 2022).

### 4.6 Phase diagrams: Comparing FH, Kyeremateng and PC-SAFT models

**Fig. 12:**
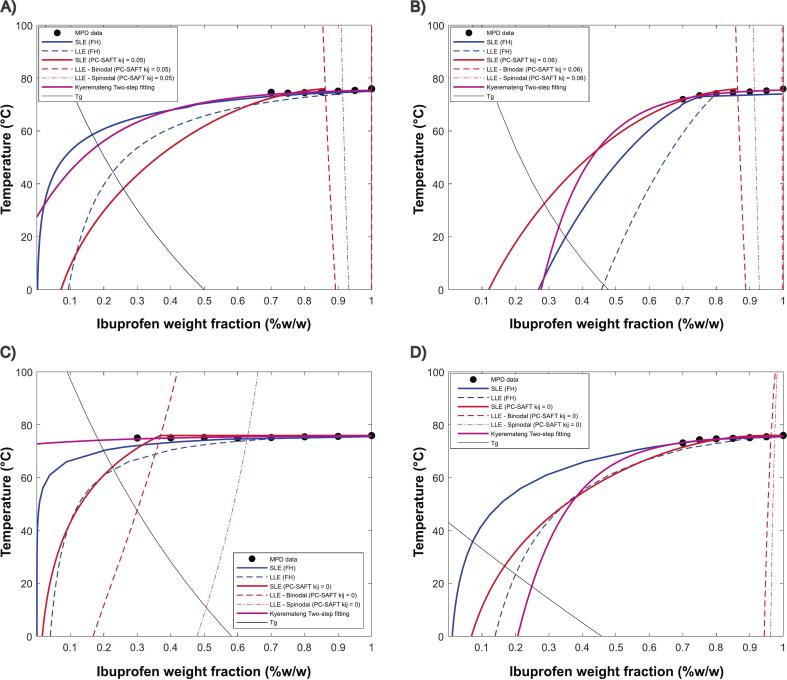
Phase diagrams of binary blends (A) KOL VA64, (B) KOL 17PF, (C) HPMCAS and (D) EPO, comparing experimental data, SLE and LLE curves modeled using FH, Kyeremateng empirical equation and PC-SAFT.

Fig. 12 presents the phase diagrams for four API–polymer binary mixtures, constructed using experimentally observed melting point depression and extrapolated using FH, fitted and unfitted PC-SAFT, and the Kyeremateng two-step fitting method. The curves indicate different solubility thresholds for the blends; however, the overall trend remains consistent: HPMCAS appears to be the least effective solvent, while the other three polymers exhibit better solubilizing capabilities.

The FH model is known to have limitations, particularly its inability to capture specific intermolecular interactions such as hydrogen bonding or ionic interactions. Additionally, its extrapolation is based on a linear relationship between the interaction parameter (χ) and the inverse of temperature, which restricts the depth of information that can be extracted and could limit the accuracy of the resulting phase diagrams (Mathers et al., 2021). While several studies support the use of FH (Tian et al., 2018a; Klueppelberg et al., 2023), others have highlighted its limitations particularly relevant to amorphous solid dispersions (ASDs), where hydrogen bonding plays a significant role (Anderson, 2018b). In addition, Kyeremateng empirical equation can be useful for screening different polymers and quickly defining a design space for HME (Kyeremateng et al., 2022). However, it is based solely on fitting experimental points and with no correlation with a thermodynamic framework, which does not enable the calculation of LLE zones, important for ASD stability prediction.

In contrast, PC-SAFT is designed to better account for molecular size differences and intermolecular interactions, resulting in more detailed and informative phase diagrams. Unlike FH, PC-SAFT predicts a broader metastable region for KOLVA64, KOL17PF, and EPO, indicating the potential for higher drug loadings, an observation consistent with the findings of Tian et al. (2020) where formulations containing up to 65 % IBU were successfully developed using EPO, despite concerns over low glass transition temperatures affecting processability. Furthermore, PC-SAFT consistently indicates that HPMCAS is a limited solvent for IBU, regardless of the number of data points or interaction parameters used (Iemtsev et al., 2020). Similar work described by Mathers et al. (2021) argues that uncertainties around FH calculations and fitting parameters interpretability from Kyeremateng empirical equation would place PC-SAFT as a more robust methodology to predict API–polymer solubility. However, a common feature among these methods is their sensitivity to the chosen variables, such as melting on-set, end-set, or peak temperature, as well as the methodology used (e.g., heating rate) and the selected data interval.

## 5 Conclusions

This study employed both hybrid and empirical modeling approaches using DSC-based methods to assess the solubility and miscibility of IBU with 4 polymers. As expected, group contribution methods based on Hildebrand and Hansen solubility parameters (Fedors, Hoftyzer-van Krevelen, and Justin-Breitkreutz) produced variations in calculated solubility values. Nevertheless, based on the general rule of thumb (Δδ < 7 MPa^½^), all blends were classified as soluble and miscible at first. Similarly, Bagley plots indicated miscibility across the blends, though borderline values were observed for HPMCAS (R_av_ = 4.47 and 4.01) and EPO (R_av_ = 1.78 and 5.34) with results varying depending on the methodology used (HVK and JB, respectively). Our novel attempt to evaluate χ derived from GC at high temperatures showed poor correlation with experimental data, limiting their reliability for solubility predictions beyond initial screening agreeing with still criticism of the lack thermodynamic correlation elsewhere (Anderson, 2018b).

Despite being the more widely used model, FH presents crucial drawbacks. As shown in Eq. 11, FH extrapolation assumes a linear relationship between χ and 1/T, which may not always hold true, particularly in complex systems where molecular interactions deviate from ideal behavior. Moreover, it fails to capture specific molecular interactions, which are crucial to understanding ASDs (Fig. 12**)**. Further limitations of FH modeling relate to its empirical nature, as parameters A and B are derived from experimental data. Consequently, χ is only predictive within the experimental conditions used to determine these parameters, restricting its applicability at temperatures significantly different from those studied. For instance, Tian et al., 2020 have identified a higher stable drug loading for IBU-EPO conducting similar experiments, but with slightly different parametrization. Kyeremateng predictions using a ‘Two-Steps’ fitting process, were considered more accurate to experimental data, deviating from its primary intended use (Kyeremateng et al., 2014). Moreover, limited information is extracted, because only SLE curves are obtained and with poor correlation between fitting parameters and a thermodynamic framework.

PC-SAFT is a promising approach which leverages from a rigorous physics-based framework to capture intermolecular interactions. Pure predictions have provided satisfactory approximations for HPMCAS and EPO, whereas fitted parameters were used to improve predictions for KOLVA64 and KOL17PF. It could be debated that with refined initial interaction parameters – dispersion energy parameter (ε_i_/k), association energy parameter (ε_iassoc/k_) – obtained from larger data sources, the pure predictions could be initially accurate, disregarding the need of fitting.

The literature on drug-polymer solubility and miscibility highlights ongoing debates and diverse methodologies (Thakore et al., 2021), which, while enriching understanding, lack a unified framework. This absence of standardization hinders the direct translation of theoretical and experimental insights into regulatory guidelines, slowing product development and increasing uncertainty in formulation design (Moseson et al., 2024). Moreover, inconsistencies between different approaches, ranging from solubility parameter-based predictions to thermodynamic modeling, underscore the need for a systematic, validation-driven methodology.

Through the work presented, we have contributed to the evolving landscape of ASD modeling by providing additional evidence on IBU solubility and miscibility with four polymers. By integrating both hybrid and empirical approaches, we highlighted the strengths and limitations of commonly used methodologies. Advancing the application of thermodynamic models, such as PC-SAFT, alongside experimental validation will help bridge the gap between theoretical predictions and practical formulation development, ultimately fostering greater maturity in ASD modeling supporting the digital transformation essential to the future of this industry.

## CRediT authorship contribution statement

**Matheus de Castro:** Writing – original draft, Methodology, Data curation, Visualization, Formal analysis, Conceptualization. **Ana Sara Cordeiro:** Supervision, Writing – review & editing. **Mingzhong Li:** Writing – review & editing, Supervision. **Christian Lübbert:** Formal analysis, Writing – review & editing. **Catherine McColl:** Writing – review & editing. **Jatin Khurana:** Writing – review & editing. **Mark Evans:** Writing – review & editing. **Walkiria S. Schlindwein:** Writing – review & editing, Project administration, Supervision, Funding acquisition.

## Funding

This work was financially supported by 10.13039/501100000601De Montfort University and Reckitt Benckiser (project number: 608587).

## Declaration of competing interest

The authors declare no conflict of interest.

## Data Availability

Data will be made available on request.

## Appendix A Supplementary data

Supplementary material Image 1
