# Supplementary material for: Advancing amorphous solid dispersions through empirical and hybrid modeling of drug–polymer solubility and miscibility: A case study using Ibuprofen
Source: Int J Pharm X. 2025 Aug 9;10:100373. doi: 10.1016/j.ijpx.2025.100373 (PMC12362086; doi:10.1016/j.ijpx.2025.100373)
Supplement: Supplementary file 1 — Supplementary material [file mmc1.docx]

***Supplementary material***

**Advancing Amorphous Solid Dispersions Through Empirical and Hybrid Modeling of Drug–Polymer Solubility and Miscibility: A case study using Ibuprofen**

Matheus de Castro^a*^,

Ana Sara Cordeiro^a^,

Mingzhong Li^a^,

Christian Luebbert^b^,

Catherine McColl^c^,

Jatin Khurana^c^,

Mark Evans^c^,

Walkiria S. Schlindwein^a^

a Leicester School of Pharmacy, De Montfort University, Leicester LE1 9BH, UK

b amofor GmbH, Otto-Hahn-Str. 15, 44227 Dortmund, Germany

c Reckitt Benckiser, Dansom Lane, Hull, HU8 7DS, United Kingdom

*Corresponding authors: Matheus de Castro ([P2827923@my365.dmu.ac.uk](mailto:P2827923@my365.dmu.ac.uk)) and Walkiria Schlindwein ([wss@dmu.ac.uk](mailto:wss@dmu.ac.uk)), Leicester School of Pharmacy, Leicester LE1 9BH, UK, England.

**Abstract**

In this supplementary section we report additional results for:

**Table S1**. Solubility parameter data calculated from Fedors, Hoftyzer-Van Krevelen (HVK) and Justin-Breitkreutz (JB).

**Table S2.** IBU–KOL VA64 melting depression data (T_end-set_) extracted using heating rates (1 and 5°C).

**Table S3.** IBU-KOL 17PF melting depression data (T_end-set_) extracted using heating rates (1 and 5°C).

**Table S4**. IBU–HPMCAS melting depression data (T_end-set_) extracted using heating rates (1 and 5°C).

* Denotes experiments that were not conducted, since 1°C experiments were conducted using broader ranges (10% drug loading steps).

**Table S5.** IBU–EPO melting depression data (T_end-set_) extracted using heating rates (1 and 5°C). Dashes (–) denotes broad peaks.

**Table S6.** Kyemerateng fitting parameters (A, b and C) from One-Step and Two-Steps fitting.

**Table S7.** AARD (%) comparison between MPD data and predictions using FH, Kyeremateng (One and Two steps fitting), PC-SAFT (k_ij_=0) and fitted PC-SAFT (k_ij_ ≠ 0).

**Table S8.** ARD and MRD comparison for modelling using GT with Simha-Boyer rule, GT with fitted k and Kwei with fitted k and calculations.

**Table S9.** Thermodynamic parameters A and B from binary blends.

**Fig. S1**. DSC data at different API-polymer compositions, glass transition temperatures (1 °C/min). A) KOL VA64. B) KOL 17PF. C) HPMCAS. D) EPO.

**Table S1**. Solubility parameter data calculated from Fedors, Hoftyzer-Van Krevelen (HVK) and Justin-Breitkreutz (JB).

|  |  | **δ_d_ (MPa^1/2^)** | **δ_p_ (MPa^1/2^)** | **δ_h_ (MPa^1/2^)** | **δ_t_ (MPa^1/2^)** | **δ_v_(MPa^1/2^)** | **R_av_** | **χ** |
| --- | --- | --- | --- | --- | --- | --- | --- | --- |
| **Fedors** | IBU |  |  |  | 20.91 |  |  |  |
|  | KOL VA64 |  |  |  | 22.91 |  |  | 0.32 |
|  | KOL 17PF |  |  |  | 23.90 |  |  | 0.71 |
|  | HPMCAS |  |  |  | 24.28 |  |  | 0.90 |
|  | EPO |  |  |  | 24.69 |  |  | 1.13 |
| **HVK** | IBU | 17.85 | 2.20 | 7.15 | 19.35 | 17.99 | 0.00 |  |
|  | KOL VA64 | 19.19 | 0.50 | 9.74 | 21.53 | 19.20 | 2.86 | 0.37 |
|  | KOL 17PF | 20.44 | 1.51 | 9.59 | 22.63 | 20.50 | 3.50 | 0.85 |
|  | HPMCAS | 19.28 | 2.28 | 11.39 | 22.66 | 19.41 | 4.47 | 0.86 |
|  | EPO | 17.35 | 0.32 | 8.81 | 19.46 | 17.35 | 1.78 | 0.00 |
| **JB** | IBU | 16.59 | 4.27 | 8.66 | 19.2 | 17.13 | 0.00 |  |
|  | KOL VA64 | 16.70 | 0.44 | 12.06 | 20.61 | 16.71 | 3.43 | 0.16 |
|  | KOL 17PF | 18.74 | 1.24 | 10.88 | 21.71 | 18.78 | 2.77 | 0.50 |
|  | HPMCAS | 17.88 | 1.02 | 4.73 | 19.52 | 17.91 | 4.01 | 0.01 |
|  | EPO | 12.09 | 0.20 | 10.44 | 15.97 | 12.09 | 5.34 | 0.82 |

**Table S2.** IBU–KOL VA64 melting depression data (T_end-set_) extracted using heating rates (1 and 5°C).

| **Mass fraction** | **T_end-set_** (1°C/min) | **T_g_** (1°C/min) | T**_end-set_** (5°C/min) | **T_g_** (5°C/min) |
| --- | --- | --- | --- | --- |
| 1 | 75.93 ± 0.10 | -44.68 ± 0.24 | 77.92 ± 0.04 | -43.57 ± 0.25 |
| 0.95 | 75.32 ± 0.07 | -41.63 ± 0.34 | 77.34 ± 0.07 | -42.04 ± 1.03 |
| 0.90 | 75.01 ± 0.05 | -39.59 ± 0.25 | 77.09 ± 0.04 | -40.23 ± 0.19 |
| 0.85 | 74.80 ± 0.04 | -37.15 ± 0.23 | 76.58 ± 0.11 | -34.55 ± 1.10 |
| 0.80 | 74.50 ± 0.16 | -28.46 ± 3.31 | 76.43 ± 0.17 | -32.01 ± 1.39 |
| 0.75 | 74.30 ± 0.13 | -29.48 ± 0.44 | 76.27 ± 0.27 | -26.61 ± 2.31 |
| 0.70 | 74.59 ± 0.10 | -24.77 ± 0.83 | 76.29 ± 0.03 | -27.23 ± 2.38 |
| 0.50 | – | 0.94 ± 2.40 | 75.84 ± 0.22 | 7.04 ± 6.25 |
| 0.40 | – | 15.39 ± 1.18 | – | 25.19 ± 1.07 |
| 0.30 | – | 33.01 ± 2.35 | – | 45.80 ± 1.40 |

**Table S3.** IBU-KOL 17PF melting depression data (T_end-set_) extracted using heating rates (1 and 5°C).

| **Mass fraction** | **T_end-set_** (1°C/min) | **T_g_** (1°C/min) | T**_end-set_** (5°C/min) | **T_g_** (5°C/min) |
| --- | --- | --- | --- | --- |
| 1 | 75.93 ± 0.10 | -44.68 ± 0.24 | 77.92 ± 0.04 | -43.57 ± 0.25 |
| 0.95 | 75.23 ± 0.02 | -42.32 ± 0.09 | 77.13 ± 0.10 | -41.83 ± 0.25 |
| 0.90 | 74.85 ± 0.03 | -39.78 ± 0.23 | 76.61 ± 0.04 | -39.30 ± 0.31 |
| 0.85 | 74.55 ± 0.02 | -37.13 ± 0.23 | 76.13 ± 0.04 | -35.47 ± 0.14 |
| 0.80 | 74.20 ± 0.07 | -32.71 ± 0.13 | 75.64 ± 0.12 | -29.53 ± 0.31 |
| 0.75 | 73.45 ± 0.04 | -27.34 ± 0.08 | 75.16 ± 0.21 | -24.58 ± 0.17 |
| 0.70 | 72.01 ± 0.24 | -21.44 ± 1.21 | 74.32 ± 0.91 | -20.53 ± 3.67 |
| 0.50 | – | 1.61 ± 0.99 | – | 6.40 ± 5.24 |
| 0.40 | – | 9.38 ± 0.69 | – | 13.27 ± 1.21 |
| 0.30 | – | 26.97 ± 1.02 | – | 29.56 ± 5.57 |

**Table S4**. IBU–HPMCAS melting depression data (T_end-set_) extracted using heating rates (1 and 5°C). * denotes experiments that were not conducted, since 1°C experiments were conducted using broader ranges (10% drug loading steps).

| **Mass fraction** | **T_end-set_** (1°C/min) | **T_g_** (1°C/min) | T**_end-set_** (5°C/min) | **T_g_** (5°C/min) |
| --- | --- | --- | --- | --- |
| 1 | 75.93 ± 0.10 | -44.68 ± 0.24 | 77.92 ± 0.04 | -43.57 ± 0.25 |
| 0.95 | * | * | 77.29 ± 0.21 | -43.62 ± 0.26 |
| 0.90 | 75.87 ± 0.05 | -42.44 ± 0.61 | 77.94 ± 0.37 | -42.87 ± 0.64 |
| 0.85 | * | * | 77.21 ± 0.23 | -41.98 ± 0.73 |
| 0.80 | 75.59 ± 0.06 | -35.58 ± 0.90 | 77.10 ± 0.09 | -36.94 ± 0.69 |
| 0.75 | * | * | 77.69 ± 0.28 | -35.58 ± 1.25 |
| 0.70 | 75.51 ± 0.02 | -31.72 ± 0.57 | 77.57 ± 0.26 | -32.47 ± 0.54 |
| 0.60 | 75.24 ± 0.04 | -3.35 ± 9.96 | * | * |
| 0.50 | 75.20 ± 0.02 | -4.34 ± 1.05 | 77.30 ± 0.14 | -14.58 ± 1.87 |
| 0.40 | 75.11 ± 0.01 | 33.45 ± 6.68 | 76.94 ± 0.10 | 24.36 ± 2.48 |
| 0.30 | 74.57 ± 0.69 | 79.30 ± 2.48 | 76.43 ± 0.06 | 71.93 ± 2.80 |

**Table S5.** IBU–EPO melting depression data (T_end-set_) extracted using heating rates (1 and 5°C). Dashes (–) denotes broad peaks.

| **Mass fraction** | **T_end-set_** (1°C/min) | **T_g_** (1°C/min) | T**_end-set_** (5°C/min) | **T_g_** (5°C/min) |
| --- | --- | --- | --- | --- |
| 1 | 75.93 ± 0.10 | -44.68 ± 0.24 | 77.92 ± 0.04 | -43.57 ± 0.25 |
| 0.95 | 75.45 ± 0.08 | -42.77 ± 0.08 | 77.66 ± 0.09 | -42.28 ± 0.23 |
| 0.90 | 75.10 ± 0.03 | -40.49 ± 0.17 | 76.72 ± 0.14 | -39.63 ± 0.60 |
| 0.85 | 74.84 ± 0.01 | -35.90 ± 0.75 | 75.58 ± 0.35 | -36.25 ± 0.90 |
| 0.80 | 74.64 ± 0.02 | -30.93 ± 0.30 | 74.88 ± 0.16 | -29.88 ± 0.54 |
| 0.75 | 74.32 ± 0.07 | -25.03 ± 0.14 | 74.40 ± 0.21 | -23.86 ± 0.27 |
| 0.70 | 73.10 ± 0.10 | -17.33 ± 1.10 | 73.67 ± 0.23 | -17.73 ± 1.46 |
| 0.50 | – | 2.92 ± 0.42 | – | 3.43 ± 0.25 |
| 0.40 | – | 6.56 ± 0.32 | – | 6.29 ± 0.40 |
| 0.30 | – | 8.12 ± 0.41 | – | 7.59 ± 0.45 |

**Table S6.** Kyemerateng fitting parameters (A, b and C) from One-Step and Two-Steps fitting.

|  | **One-Step Fitting** | | | **Two-Step Fitting** | | |
| --- | --- | --- | --- | --- | --- | --- |
| **Blends** | **A** | **b** | **C** | **A** | **b** | **C** |
| IBU-VA64 | 36.6313 | -0.05 | -0.5325 | 45.2715 | -0.0438 | 0.0023 |
| IBU-17PF | 67.75 | -0.05 | -0.1181 | 536.71 | -0.0707 | -0.0135 |
| IBU-HPMCAS | 5.1018 | -0.05 | -0.2306 | 3.1769 | -0.0296 | 0.0042 |
| IBU-EPO | 98.20 | -0.05 | 0.3809 | 308.005 | -0.0678 | -0.0103 |

**Table S7.** AARD (%) comparison between MPD data and predictions using FH, Kyeremateng (One and Two steps fitting), PC-SAFT (k_ij_=0) and fitted PC-SAFT (k_ij_ ≠ 0).

| **Blend** | **FH** | **Kyemerateng (One-Step fitting)** | **Kyemerateng (Two-Steps fitting)** | **PC-SAFT (k_ij_=0)** | **PC-SAFT (k_ij_ ≠ 0)** |
| --- | --- | --- | --- | --- | --- |
| IBU-VA64 | 0.32 | 0.24 | 0.24 | 4.30 | 1.50 |
| IBU-17PF | 0.99 | 0.44 | 0.16 | 4.22 | 0.86 |
| IBU-HPMCAS | 0.96 | 0.13 | 0.11 | 1.26 | – |
| IBU-EPO | 0.12 | 0.20 | 0.16 | 0.97 | – |

**Table S8.** ARD and MRD comparison for modelling using GT with Simha-Boyer rule, GT with fitted k and Kwei with fitted k and calculations.

| **Parameter** | **GT -Simha-Boyer**  **AARD (%)** | **GT -Simha-Boyer**  **ARD (%)** | **GT-Fitted K**  **AARD (%)** | **GT Fitted k**  **ARD (%)** | **Kwei (fitted q)**  **AARD (%)** | **Kwei (fitted q)**  **ARD (%)** |
| --- | --- | --- | --- | --- | --- | --- |
| IBU-KOL VA64 | 2.67 | 2.67 | 0.89 | 0.43 | 0.91 | 0.33 |
| IBU-KOL 17PF | 4.77 | 4.77 | 0.75 | 0.10 | 0.76 | 0.14 |
| IBU-HPMCAS | 4.62 | 1.77 | 4.65 | 1.87 | 4.47 | 1.39 |
| IBU-EPO | 1.44 | -0.61 | 1.33 | 0.41 | 1.33 | 0.40 |

**Table S9.** Thermodynamic parameters A and B from binary blends.

| **Blend** | **A** | **B** |
| --- | --- | --- |
| IBU-KOL VA64 | -17.06 | 6138.8 |
| IBU-KOL 17PF | -0.94 | 563.6 |
| IBU-HPMCAS | -122.3 | 42869 |
| IBU-EPO | -10.43 | 3834.3 |


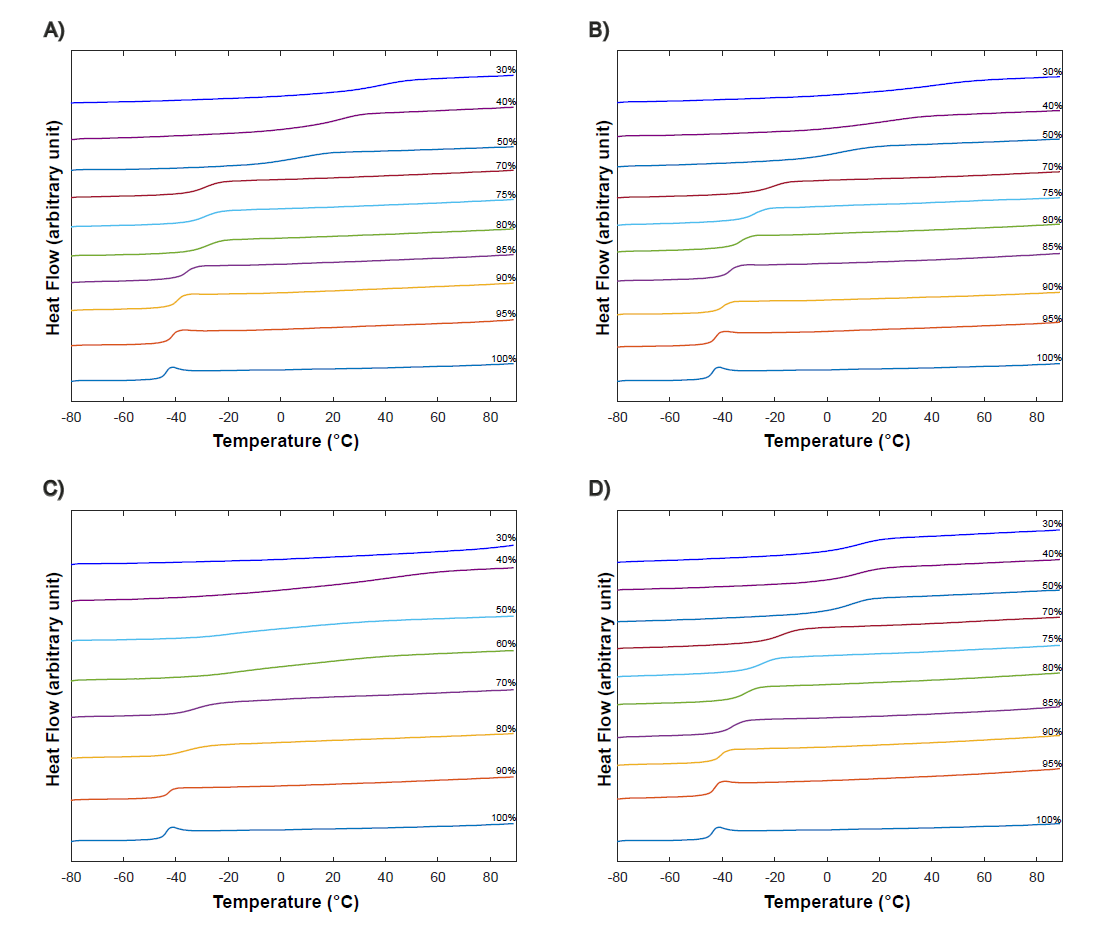


**Fig. S1**. DSC data at different API-polymer compositions, glass transition temperatures (1 °C/min). A) KOL VA64. B) KOL 17PF. C) HPMCAS. D) EPO.
